# Are prime numbers special? Insights from the life sciences

**DOI:** 10.1186/s13062-022-00326-w

**Published:** 2022-05-27

**Authors:** Maria Loconsole, Lucia Regolin

**Affiliations:** Department of General Psychology, University of Padua, Padua, Italy

**Keywords:** Prime numbers, Perceptual grouping, Quasi-experiments, Domestic chick

## Abstract

Prime numbers have been attracting the interest of scientists since the first formulation of Euclid’s theorem in 300 B.C. Nowadays, physicists and mathematicians continue to formulate new theorems about prime numbers, trying to comprehensively explain their articulated properties. However, evidence from biology and experimental psychology suggest that prime numbers possess distinctive natural properties that pre-exist human grasping. The present work aims at reviewing the existing literature on prime numbers in the life sciences, including some recent experimental contributions employing newly hatched domestic chicks as animal model to test for spontaneous mechanisms allowing discrimination of primes from non-primes. Our overarching goal is that of discussing some instances of prime numbers in nature, with particular reference to their peculiar, non-mathematical, perceptual properties.

## Background

In his 1985 sci-fi novel, the American scientist Carl Sagan imagined a first contact between the humankind and an extra-terrestrial civilisation, where the aliens sent a message that consisted of a sequence of prime integers [1]. The protagonist deciphered the message and described prime numbers as a universal system of communication, as well as an expression of an “intelligent mind”. Besides the complex narrative plot of the book, Sagan instilled in readers’ minds a fascination for prime numbers and for their uniqueness. One of the greatest philosophical questions that aroused curiosity in experts, as well as in the common men for thousands of years is whether we invented mathematic or rather just “discovered” it. In other words, did we create mathematical rules to better comprehend the world? Alternatively, are mathematical properties embedded in nature and were they subsequently translated into formulas and laws by acculturated humans? This work does not aim at answering to these questions (nor we believe a final answer is there, yet), but rather aims at stimulating a debate on this intriguing topic, tapping into anecdotal and laboratory works from the biological sciences with a focus on the case of prime numbers.

## Main text

Prime numbers have attracted the interest of scientists since the first formulation of Euclid’s theorem in 300 B.C. These are integer positive numbers that do not result by the product of other numbers (i.e., they can only be divided by 1 and themselves). The product of two prime numbers is a composite number. Thus, prime numbers could be considered as the building blocks of natural numbers, as atoms are the building blocks of matter in chemistry or physics [2]. Nowadays physicists and mathematicians continue to formulate new theorems about prime numbers, trying to explain comprehensively their articulated properties. Prime numbers have been implemented in several aspects of human life, e.g., they are at the basis of modern computational security and cryptography. Interestingly, prime numbers are not only to be found in artificial human-made systems, but there are examples of primes also in the natural world.

## Prime numbers in nature

### The case of the American cicadas

Some mathematical concepts can be found in natural phenomena, as in the case of the Fibonacci sequence, and the associated Golden ratio, which can be found in several instances of biological settings, e.g., the branches of a tree, the leaves on a stem, or the seeds on the sunflower [3, 4]. The most striking case of prime numbers embedded in an ecological context is probably represented by American cicadas (*Magicada* spp.). In these periodical cicadas, the larvae are buried in the ground and emerge every 13 or 17 years to turn into adults [5, 6]. It has been hypothesized that the emergence of nymphs according to such an improbable time lapse would reduce the risk of predation by minimizing the chances of encountering predators with a synchronized life cycle, and thus increasing the chances of successful reproduction. For instance, a cicada that emerges every 12 years will synchronize with all predators having a life cycle of 2-, 3-, 4-, 6- or 12- years, whereas emerging every 13 years strongly reduces such a chance. Moreover, as the chances of surviving (and consequently mating) is dependent on the exact synchronization of their life cycle, interbreeding should also be avoided, as it might cause a loss of the precise timing. As both species largely share the same habitat, interbreeding might represent an actual risk. However, as they both rely on a prime-based life cycle their simultaneous emergence takes place only once every 221 years (i.e., 13 × 17). Would their life cycles be based on composite numerosities, they would overlap as many times as the common multiples of such numerosities. The case of the American cicadas exemplifies the presence of a mathematical concept in the absence of the need for the animal to have any grasping or reasoning on such concept (although some sense of number has indeed been described in invertebrates [7]). This is rather a neat example of a mechanism shaped by natural selection which exploits the property of non-factorization of prime numbers.

### The biological mechanism for the prime-number emergence cycle

In spite of the clear evolutionary advantage, it is difficult to exactly identify the mechanism by which cicadas achieve such a unique combination of periodicity, synchrony, and extraordinary long developmental period. This has to rely on a high level ecological mechanism, to the point that some authors suggested that periodical cicadas have reached an adaptive peak [8]. To our knowledge, there is no conclusive evidence to date that can explain the fine physiological timing mechanism reported in the *Magicada*. Basilar organic processes, such as circadian rhythm, biological clock, or internal and external triggers, do not seem sufficient to account for its precision with respect to the capability to process and respond to a time window as large as 13 or 17 years. The”periodical cicadas problem” [5, 8] is a unique and much debated phenomenon in biology. The possibility of prime numbers having some peculiar features that support such a mechanism is fascinating, and it captivates scientists from several disciplines other than the biological sciences, such as mathematic and philosophy.

## Evidence of prime numbers identification in humans

### Single-case studies and quasi-experimental evidence

Oliver Sacks’ book *The Man Who Mistook His Wife For A Hat* (1985) contains probably the most famous report of prime numbers identification and generation in people with extremely poor (or even absent) mathematical skills. In particular, Sacks recounted of two twins with Autism Spectrum Disorder (ASD) and *Savant* syndrome who could generate multiple digit prime numbers in some sort of playful challenge, i.e., each would respond to the other with a higher prime number. According to Sacks, when he challenged them by taking part to the game (with the aid of a prime numbers table) the twins generated prime numbers of up to 10-digits [9]. However, it must be noted that this report suffered from some criticisms, which never questioned the twins’ ability but highlighted the need of scaling them down [10–12]. For instance, the twins generated very high prime numbers that exceeded Sacks’ number table, making it impossible to verify the correctness of such responses. Another interesting case is the one reported by Hermelin and O’Connor (1990). The authors described the performance of Michael, a young man with ASD, who could factorize prime numbers greater than 10,000 with a 70% accuracy (compared to a mathematically trained control subject who scored only 40% accuracy and slower response times). With numbers lower than 10,000 Michael’s accuracy further increased, reaching 85% [13]. In a subsequent study Anderson and colleagues expanded on this first research, trying to assess the mechanism underlying Michael’s abilities by adopting a more strict experimental approach [14]. They compared Michael’s reaction times in a prime number identification task to a control group that had been instructed to apply specific mathematical strategies. In one condition, the controls were trained to use Eratosthenes’ sieve; in another condition they used a simple memory strategy, where they had to learn and remember a list of prime numbers in growing order. Even though Michael was in all cases significantly faster, the pattern of his response times matched that of the group using Eratosthenes’ sieve. This consists in dividing the target number by all the prime numbers less than, or equal to, the target itself. If the target number cannot be divided by any of these numbers without a remainder, then it is a prime number. This study aimed at providing indirect evidence of a mechanism at the basis of prime number identification, which could be inferred by some correspondence in the kind of errors made and in the progression of response times with increasing numerosity between the tested subject and the control group. Yet, this does not explain why Michael’s reaction times (although following a similar trajectory) were significantly faster than those of the controls (on average Michael responded in 35–40 s, while the control group required on average 50 s), nor does it explain how Michael could have relied on such a complex algorithm in the absence of attested mathematical skills.

**Fig. 1:**
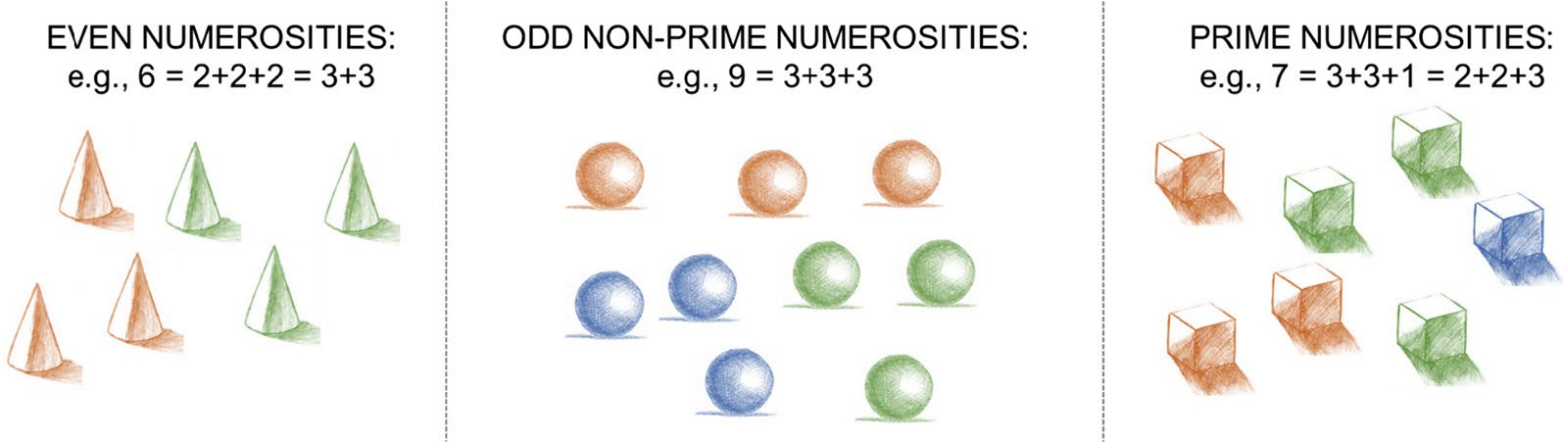
Grouping (by colour) of: even, odd non-prime, and prime numerosities. In this example, it is possible to see how, in line with what hypothesized by Welling, one can visually disassemble composite numerosities into same-sized subsets, but the same is not true for prime numerosities, which will always result in at least one subgroup being of a different numerosity. Here elements are grouped by colour, but it is possible to obtain the same result employing other grouping strategies based on Gestalt principles, such as shape similarity or spatial proximity

Besides the use of Eratosthenes’ sieve, another interesting hypothesis is the one suggested by Welling (1994), based on the idea that subjects with ASD possess a system that enables them to mentally represent numbers as visual images and manipulate them in a way that resembles mathematical computations [15]. In line with this reasoning, prime numbers could be identified according to a visual grouping strategy that mimics mathematical factorization (Fig. 1). In fact, a composite number can always be divided into subgroups all having the same number of elements (e.g., 6 = 3 + 3 or 2 + 2 + 2, 9 = 3 + 3 + 3) whereas prime numbers will always result in at least one of the subgroups being of a different numerosity (e.g., 7 = 3 + 3 + *1*, or 2 + 2 + *3*, or 3 + 4 + *1*, etc.). This would be consistent with studies highlighting segmentation strategies in ASD subjects with exceptional mathematical skills [16, 17]. In fact, these subjects showed a tendency to create subsets by dividing large numbers into their factors, or into smaller chunks [16, 18].

Welling refers to the grouping of elements into same-size subsets as symmetrical grouping. This would be in line with the Gestalt principles of *pregnanz*, or good figure [19], as a symmetrical sub-setting implies a more balanced and harmonic perception. Consequently, prime numbers only allow for an asymmetrical grouping, as they will never satisfy the requirement of all subgroups having the same number of elements. This somehow resembles the idea put forward by Anderson and colleagues [14], but without the need to hypothesize the use of a mathematical algorithm. However, up to this point, Welling’s hypothesis remains merely speculative, due to the lack of experimental evidence of visual perception of large numbers, differential sensitivity to symmetrically (vs. non-symmetrically) arranged sets and lack of evidence in support of these mechanisms directly affecting prime numbers recognition.

### Visual-working memory and grouping strategies in prime number identification

Recent studies provided evidence that supports the idea of ASD subjects possessing a strong ability to visually represent and manipulate images in working memory. ASDs are often characterized by an enhanced perceptual processing ability, and some autistic individuals perform better than non-autistic controls in a variety of tasks that require visual-spatial operations and low-level perceptual processing [20, 21]. This hyper attention toward visual-spatial features could also lead to an enhanced capability in identifying regular patterns and detecting small- or large- scale units without involving high-level non-perceptual processes [20]. Pattern recognition might be linked to the process of grouping, which requires to detect a structured organization according to certain principles shared between the elements of a configuration (e.g., proximity, similarity, symmetry) [20, 22, 23]. ASD subjects have indeed demonstrated comparable or even higher performance with respect to non-ASD subjects in visual memory, visual search, and discrimination of first-order relationships. These reports can only provide indirect evidence in support of Welling’s theory; however, they could still be mentioned.

A study by Ciccione and Dehaene (2020) offers some important insights on the role of the aforementioned grouping strategies in the processing and identification of prime numbers in the normal population [22]. The authors aimed at testing cognitive strategies that could improve performance in mathematical tasks. Subjects were college students with low, medium, or high mathematical skills. They were required to enumerate sets of dots presented on a screen under different conditions. For the sake of our argument, we will consider three conditions: 1. Dots were all the same colour and randomly scattered in the space; 2. Dots were grouped in smaller subgroups (either by colour or spatial position), each subgroup being of a different numerosity for both primes and non-primes (the one we defined as asymmetrical grouping [15]); 3. Dots were grouped in smaller subgroups (either by colour or spatial position), with all the subgroups having the same number of elements (the one we defined as symmetrical grouping [15]). In this latter case, however, prime numbers were grouped as two groups of the same numerosity, and a third group of a different one, due to the impossibility of creating all equal subgroups. The accuracy and reaction times for enumerating each stimulus were recorded in the three conditions. The authors hypothesized that arrays divided into same-size subsets should be faster enumerated, as such a display facilitates a multiplicative processing (e.g., in the case of 9 elements, rather than adding one element up to each other until reaching the desired numerosity, the subjects could opt for a faster strategy such as computing three times three). In the first two conditions the authors found a well-known effect in numerical studies, namely the numerical size effect, for which when the magnitude of the set to be enumerated increases, the reaction time increases as well. However, in the multiplicative condition (i.e., when all subgroups were of the same numerosity) this effect was not matched in the case of the numbers 5, 7, and 11, i.e., all the prime numbers employed in the task. In such a case, reaction times were slower with respect to both the set preceding and the one following the prime numerosity. The authors addressed this effect by the fact that same-size subsetting was not complete with prime numbers, being one of the subgroups of a different numerosity (i.e., 5 = 2 + 2 + 1; 7 = 3 + 3 + 1; 11 = 3 + 3 + 3 + 2). In fact, this cannot be considered a pure multiplicative strategy (as it was for composite numbers) but a mix of both multiplicative (for the subgroups of the same size) and additive processes (i.e., 5 = 2 × 2 + 1; 7 = 3 × 2 + 1; 11 = 3 × 3 + 2). A similar effect was also reported in other studies, showing that participants are faster when responding to a larger (but symmetrical) number rather than to a smaller (but asymmetrical/prime) one, as for six with respect to five, or eight with respect to seven [24, 25]. The drop in performance when responding to prime numbers vs. same-sized grouped composite numbers appears irrespective of the subjects’ mathematical skills, even though there is a general effect of expertise (i.e., the higher the mathematical skills, the better the performance). This might suggest that perceptual asymmetry is a precocial information that foreruns a more thoroughly (probably based on symbolic strategies) analysis and takes place at the initial stages of processing. If this was the case, we might hypothesize that subjects who are completely naïve with respect to mathematical concepts and calculations might also exploit on such a perceptual processing to discriminate between numerosities that allow for symmetrical grouping from those than never allow creating all same-size subsets (i.e., prime numbers).

## Experimental studies on prime numbers discrimination in a non-human model

### The domestic chicken as a model species

The newly hatched domestic chicken allows to study predisposition and spontaneous strategies/mechanisms. Experimental paradigms can exploit on these birds’ early learning abilities, as well as their precocial abilities in perceptual and cognitive processing. During their very first hours of life, chicks exhibit a strong tendency to explore the environment and learn its regularities [26]. Domestic chicks are endowed with a variety of numerical processing abilities, such as discrimination of small and large quantities [27, 28], proto-arithmetic calculation [29], and ordinal reasoning [30]. They are also susceptible to a spontaneous crossmodal representation of space and number [31], and their numerical performance benefits from passively induced grouping strategies [32]. Chicks possess fine perceptual processing skills and respond to optical illusions according to Gestalt principles [33]. Lastly, chicks can process and respond to information about object symmetry [34–36]. Experimental evidence has been recently put forward of prime vs. non-prime number discrimination in newly hatched domestic chick. This to our knowledge is at present the only study carried out in non-humans on this topic.

### An experimental study on prime numbers discrimination in baby chicks

**Fig. 2:**
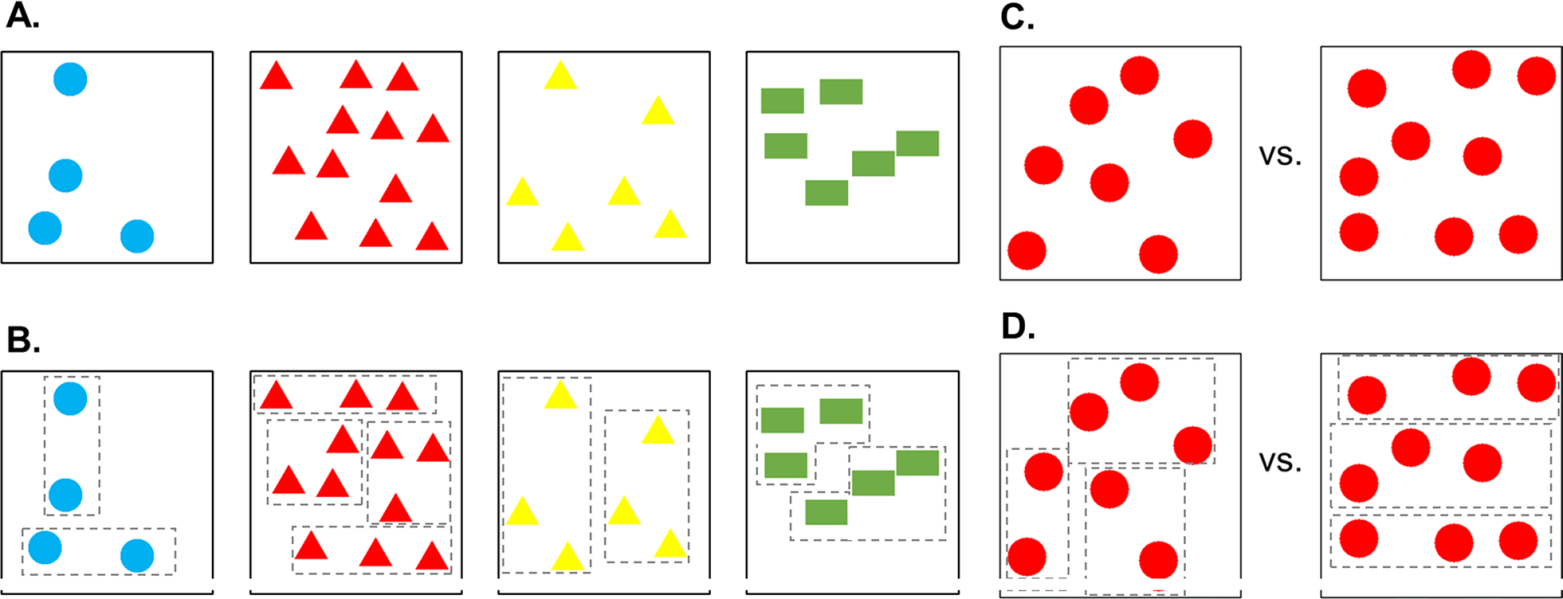
**A** Familiarization stimuli. Each subject saw a random sequence of 360 computer presented stimuli depicting an even numerosity. Here is an example of four different combinations of: even numerosity, colour and shape of the elements. **B** Symmetrical grouping could be experienced during familiarization. Familiarization stimuli could be perceptually disassembled into same sized sets (symmetrical grouping). In this example the elements are visually grouped within dashed grey virtual lines. **C** Example of the testing comparison. At test, stimuli were presented in pairs, each pair comprising elements of the same colour and shape, but of different numerosities (i.e., 7 vs. 9 or 9 vs.11). Each pair remained visible for 10 s on a monitor and was immediately replaced by another one, a total of 30 pairs were displayed during the test. **D** Symmetrical versus asymmetrical grouping during the test. Both test numerosities, being odd, were novel with respect to the familiarization. However, numerosity 9 could be still symmetrically grouped (3 + 3 + 3) whereas 7 (in the example) and 11 would always result in at least one subgroup having a different number of elements (e.g., 7 = 2 + 2 + 3). The same also applies for 9 versus 11 (not shown in the figure)

The studies on grouping as a strategy to improve numerical performance in humans [22, 24, 25] provided some interesting insights on a visual property that might characterize prime numbers, namely the fact that these will always result in perceptually asymmetrical grouping. The aforementioned studies, highlighted subjects’ difficulty in responding to asymmetrical visual patterns when compared to symmetrical ones. Yet, this only provides indirect evidence of prime numbers possessing some characteristic perceptual features, as the hypothesis was never directly tested. A recent study tested whether newborn chicks (*Gallus gallus*) could exploit on perceptual asymmetry to discriminate prime from composite numerosities [37]. In a preliminary “familiarization” phase, day old chicks were exposed for 1 h to a sequence of computer presented stimuli, these were sets of elements. This phase started few hours after hatching, so that chicks did not have any prior experience of numerical information or perceptual symmetry. All of the sets presented during familiarization depicted even numerosities, each set being made of either 4, 6, 10 or 12 elements presented in random spatial position on the screen. Each stimulus consisted in a random combination of one of these 4 numerosities, one of 3 types of elements (rectangles, triangles, circles), and one of 4 colours (i.e., blue, red, green, yellow), and remained visible on the monitor for 10 s before being replaced by a subsequent stimulus. This way a chick experienced a total of 360 stimuli during familiarization. This procedure prompts chicks’ interest for the stimuli and allows them to learn their basic features. One hour after the end of the familiarization phase, chicks were let free in the same arena used for familiarization for 5 min during which time they could freely choose to approach and inspect either of two new sets of elements: an odd non-prime numerosity (i.e., 9) and a prime numerosity (i.e., either 7 or 11). These stimuli comprised elements made of the same colours and shapes experienced during familiarization, though both sets appeared as novel in that they depicted an odd numerosity. One of them though (the set of 9 elements), can be regarded as more familiar than the other, as odd non-prime numbers are composite, just like even numbers, and can be successfully disassembled into same-sized subsets (symmetrical grouping). It is established that chicks preferentially explore stimuli regarded as novel when exposed to stimuli resembling the familiar ones in a familiar environment [38, 39]. A preference for the prime numerosity may have emerged at test if chicks did employ grouping strategies to process and discriminate the two sets. In fact, the prime numerosity at test would constitute chicks’ very first experience of a set which can not possibly be disassembled symmetrically (Fig. 2).

Consistent with the initial hypothesis, chicks significantly spent longer by the prime numerosity at test, thus demonstrating the capability to discriminate and prefer it when compared to the non-prime odd numerosity. This preference was interpreted as a preference for the stimulus regarded as novel, as compared to the composite (even) sets experience during familiarization.

The fact that the preference appeared both when the prime numerosity was the smaller (7 vs. 9) and when it was the larger (9 vs. 11) in the comparison allowed to rule out a simple preference for the magnitude (a preference for the larger set of familiar elements has in fact been described in chicks [27, 29]). Moreover, a choice based on numerical (i.e., chicks computing and comparing the numerical size of each whole set), rather than perceptual, processing seems unlikely because of the complexity of the employed comparisons. In fact, chicks’ numerical abilities have been successfully described only for much smaller numerosities [27, 40] or with higher ratio between the two sets [28, 41]. In spite of this, the data does not provide direct evidence of the hypothesis that chicks do disassemble and compare sets into smaller and same-size subgroups.

To provide a direct test of the role of the perceptual grouping mechanism new chicks were tested employing the same procedure but with a numerically more difficult comparison, i.e., 13 versus 15 (ratio of 0.87), either without or in the presence of grouping cues. Chicks that were tested without grouping cues, underwent the same procedure described for the 7 versus 9 and 9 versus 11 experiment. For chicks in the grouping cue condition, each familiarization stimulus was made of sets comprising all three different colours, arranged to avoid cueing any perceptual grouping (i.e., stimuli of the same colour were never close to each other). At test, however, the elements of each stimulus were presented as chunked by colour: numerosity 13 was presented as instances of 5 + 5 + 3 (e.g., 5 blue, 5 red, and 3 yellow triangles), whereas 15 as 5 + 5 + 5 (e.g., 5 blue, 5 red, and 5 yellow triangles). Only chicks of the cued condition succeeded in the 13 versus 15 discrimination, while chicks that could not passively resort on the colour grouping failed, most likely due to the difficulty of processing this comparison. These data support the idea of symmetrical grouping being at the base of chick’s choice at test in the 9 versus 11 and 7 versus 9 conditions. Moreover, it offers an important insight regarding the functioning of this perceptual mechanism. In fact, even though this could represent a purely perceptual strategy, that requires no former numerical training or mathematical skills, it is subjected to a set-size or processing limitation. When exceeding a certain numerical threshold, this mechanism is no longer effective, as it is too demanding for the system to create and keep track of all the subgroups. This observation might seem in contrast with reports on ASD subjects who could discriminate numbers in the order of thousands. However, a direct comparison seems untenable. The study on chicks was focused on a direct analysis of the perceptual mechanism and its origin, thus relying on data from day-old animals in a fully controlled environmental setting. In such a condition, where chicks were naïve of any prior experience with numerosity, symmetry perception, or grouping strategies, we could observe a spontaneous mechanism that enables baby chicks to solve complex numerical discriminations via a perceptual strategy. The system of ASD-subjects for recognizing or even generating prime numbers might be rooted on analogous primitives, but it certainly features greater inter-individual variability, as subjects differed broadly in age, education, and relevant prior experiences.

## Future directions and experimental studies on mathematically naïve subjects

### Studies on the domestic chick

Further studies on non-human models are fundamental to reach a deeper comprehension of the perceptual grouping mechanism that domestic chicks seem to use for discriminating prime numbers. A crucial point to be addressed in future studies is whether the reported preference for prime numbers did arise because of the familiarization on even numerosities (i.e., the hypothesized response to novelty) or whether a spontaneous preference for asymmetrical configurations was there to start with. The familiarization is crucial to direct chicks’ attention to the artificial stimuli employed at test, hence enabling their behavioural response at test, and minimizing any freezing or avoidance reactions chicks would naturally show in front of totally unfamiliar objects. Nevertheless, the stimuli used during familiarization could be experimentally manipulated, for example chicks familiarized with prime (instead of even) numerosities should regard new instances of primes at test as familiar. Would these chicks react by approaching the novel (composite) stimulus? Or would they still approach the prime, pointing at a spontaneous predisposition for asymmetrical grouping?

Also, the role of the superimposed perceptual grouping (for example by means of the use of different colours for identifying the subsets [37]) should be clarified to ascertain how it affects the perceived asymmetry. In fact, in the case of Loconsole et al., the passively induced grouping was congruent, in that the composite number was presented as symmetrically grouped and the prime number as asymmetrically grouped. It is still unknown how baby chicks would react to incongruencies, as in the case of a composite number (e.g., 9) presented as asymmetrically grouped (e.g., into sets of 3, 4 and 2 elements). This would allow to understand whether the supposed perceptual mechanism acts by detecting any salient asymmetry in the stimuli or whether it is tuned more specifically to the numerical properties of the sets. In the latter case chicks may still prefer a prime numerosity to an asymmetrically grouped composite numerosity. Indeed, some mechanism to discriminate sets was successfully employed by the chicks also in the absence of any superimposed grouping in the 7 versus 9 and 9 versus 11 comparisons.

These are only some of the issues worth of investigation in the near future.

### Other non-human animal models

The study on the domestic chicks represents an inspiring starting point for unveiling the presence of a perceptual mechanism based on the detection of symmetrical patterns. The fact that the evidence comes from a non-mammalian species is particularly insightful, as it suggests that the mechanism might be widespread among vertebrates, and it might bear significant ecological value. Yet, from a comparative approach, further investigation is required. In particular, extending the evidence to different mammalian species, to better understand to which extent this mechanism is available and to pinpoint its signature features. When considering the studies on humans, participants were described as spontaneously motivated to generate or recognize prime numbers (e.g., to play a game, in the case of Sacks [9]), alternatively, subjects were explicitly instructed by the experimenters to do so (as for O’Connor and colleagues [13, 14]). Studies on non-human species would be extremely insightful and we deem they are highly needed. Data from a non-human model (for instance, non-human primates) may not necessarily enlighten similarities with the human performance, especially considering that there are some cases in which primates see the world differently than humans, and this often includes perception of visual phenomena [42, 43]. The study on chicks exploited some species-specific characteristics (see “The domestic chicken as a model species” section) to observe a response to prime numerosities, which make the chicks a highly suitable model but at the same time questions generalizability of the findings. Hence, the study of different non-precocial avian species would be crucial. Both studies on mammals and birds, other than the relevance for a deeper comprehension of the natural perceptual features of prime numbers, could also take on great importance for further phylogenetic studies. If the data support the existence of a widespread non-mathematical mechanism for asymmetrical grouping, that can help to discriminate prime numbers via some intrinsic perceptual features, it is indeed legitimate to assume that such a mechanism might date back in time. If this were the case, we could expect to find a different response to symmetrical vs. asymmetrical sets of numbers also in fish and reptiles. Furthermore, it might also be the case of analogous mechanisms being detectable even by invertebrate species, which had to face similar environmental challenges and are already known to share many more cognitive and perceptual capabilities than previously thought with vertebrates [44–46].

### Human infants

The studies on chicks, together with some indirect evidence of prime numbers from ethology (i.e., the case of the *Magicada*) and human psychology (i.e., reports of the capability of some individuals to recognize and generate prime numbers, and the experimental evidence of a hindering effect in enumerating prime numbers), provides some critical arguments that force us to re-think the biological meaning of prime numbers. In fact, it might be the case of prime numbers not being the mere result of human culture and education but being somehow embedded in nature due to the peculiar features that characterize them. If prime numbers do possess some unique perceptual features, we may expect to observe an effect similar to that described in chicks also in our species well before of any formal acculturation, i.e., in preverbal infants. In fact, this would not be the first case of evolutionary convergence of perceptual and cognitive abilities shared by human infants and baby chicks. There is a wide literature focused on the ontogenetic origin of human knowledge that supports the existence at birth of some dedicated predisposed cognitive systems, each dealing with the representation of a specific category of basic knowledge, i.e., the so called core knowledge systems [47–49]. Such systems would be phylogenetically ancient, as they were identified in different human cultures, and in a variety of animal species and clades. Being available at birth, the core knowledge systems are considered as the building blocks onto which more complex cognitive capabilities are developed during ontogenesis. Evidence of shared core systems of knowledge between human infants and baby chicks includes grasping of naïve physics concepts [50, 51], recognition of inanimate vs animate objects [52–54], and number sense [49, 55].

Symmetry-based perceptual mechanisms for processing sets of elements may also constitute or belong to one of these building blocks of knowledge, being it available at the early stages of ontogeny (at least in the baby chick) and spontaneously employed. However, while chicks are a precocial species, human offspring is altricial. Different selective pressures have been acting on the two species, likely leading to a different emergence of traits or cognitive mechanisms. Newly hatched chicks need to be able to interact and respond to their environment from the very early moments of their life, hence fine perceptual mechanisms would be beneficial to enable them to detect patterns and regularities (to discriminate patches of food, or social objects). Analogous perceptual mechanisms were described in humans [15, 22], suggesting a low-level perceptual analysis that is not influenced by cultural or educational factors, though it is not easy to finally ascertain whether these may be an instance of evolutionary convergence or not. To tackle this issue a more direct comparison among the two species is warranted. The phylogenetic distance, together with the many differences in their ecological niche, adds further relevance to the comparison between human infants and newly hatched domestic chicks. Even though previous studies provided evidence of analogous mechanisms in other cognitive domains (e.g., social orienting [56], biological motion [52, 53], face perception [56, 57], space-number association [31, 58]), nothing is known about the existence in infants of a perceptual mechanism for grouping. Some preliminary data [59] would offer support for an analogous mechanism in four-month-old infants. Interestingly, infants, just like the newly hatched domestic chicks seem to spontaneously discriminate prime from non-prime odd numerosities. Twenty-four four-month-old infants, following familiarization with even numerosities, looked longer at sets of elements for which same-size grouping was never possible (e.g., preferring 7 to 9). If confirmed, these findings would further support the idea of a widespread biological mechanism present in distantly related species and suggest that perceptual grouping might constitute an innate predisposed cognitive system of knowledge.

## Conclusions

In the present work we covered the current evidence on the occurrence of prime numbers in animal strategies, either for species’ survival or for cognitive processing. The aim was that of stimulating critical thinking on the possibility of some properties of prime numbers being embedded in nature and inherently perceived by animals. This would be possible thank to some peculiar characteristics that define these numbers and make it possible to discriminate them from non-primes. We believe that the reported data ultimately provide some insights onto analogous mechanisms that can serve for prime number identification paving the path for future investigations aimed at uncovering the underlying biological basis. We expect this work to highlight the role of non-mathematical strategies employed in numerical tasks, with the added aim of fostering a stimulating debate on the naturalistic properties of numbers. Recalling the words of the physicist Arnoldo Penzias, "Should the Universe end tomorrow, 7 would still be a prime number" [60].

## Data Availability

Data sharing is not applicable to this article as no datasets were generated or analysed during the current study.
